# The impact of preventive screening resource distribution on geographic and population-based disparities in colorectal cancer in Mississippi

**DOI:** 10.1186/s13104-015-1352-0

**Published:** 2015-09-08

**Authors:** Fazlay S. Faruque, Xu Zhang, Elizabeth N. Nichols, Denae L. Bradley, Royce Reeves-Darby, Vonda Reeves-Darby, Roy J. Duhé

**Affiliations:** GIS and Remote Sensing Program, University of Mississippi Medical Center, Jackson, MS 39216-4505 USA; Center of Biostatistics and Bioinformatics, University of Mississippi Medical Center, Jackson, MS 39216-4505 USA; Murrah High School, Jackson, MS 39216 USA; University of Pennsylvania, Philadelphia, PA 19104 USA; GI Associates and Endoscopy Center, Jackson, MS 39202 USA; Department of Radiation Oncology, University of Mississippi Medical Center, 2500 North State Street, Jackson, MS 39216-4505 USA; Department of Pharmacology and Toxicology, University of Mississippi Medical Center, Jackson, MS 39216-4505 USA; UMMC Cancer Institute, University of Mississippi Medical Center, Jackson, MS 39216-4505 USA; Vanderbilt University, Nashville, TN 37240 USA; University of Mississippi, Oxford, MS 38677 USA

**Keywords:** Colorectal cancer (CRC), Colonoscopy, Drive-time, Geographic Information Systems (GIS), Health disparities, Screening disparities

## Abstract

**Background:**

The state of Mississippi has the highest colorectal cancer (CRC) mortality rate in the USA. The geographic distribution of CRC screening resources and geographic- and population-based CRC characteristics in Mississippi are investigated to reveal the geographic disparity in CRC screening.

**Methods:**

The primary practice sites of licensed gastroenterologists and the addresses of licensed medical facilities offering on-site colonoscopies were verified via telephone surveys, then these CRC screening resource data were geocoded and analyzed using Geographic Information Systems. Correlation analyses were performed to detect the strength of associations between CRC screening resources, CRC screening behavior and CRC outcome data.

**Results:**

Age-adjusted colorectal cancer incidence rates, mortality rates, mortality-to-incidence ratios, and self-reported endoscopic screening rates from the years 2006 through 2010 were significantly different for Black and White Mississippians; Blacks fared worse than Whites in all categories throughout all nine Public Health Districts. CRC screening rates were negatively correlated with CRC incidence rates and CRC mortality rates. The availability of gastroenterologists varied tremendously throughout the state; regions with the poorest CRC outcomes tended to be underserved by gastroenterologists.

**Conclusions:**

Significant population-based and geographic disparities in CRC screening behaviors and CRC outcomes exist in Mississippi. The effects of CRC screening resources are related to CRC screening behaviors and outcomes at a regional level, whereas at the county level, socioeconomic factors are more strongly associated with CRC outcomes. Thus, effective control of CRC in rural states with high poverty levels requires both adequate preventive CRC screening capacity and a strategy to address fundamental causes of health care disparities.

## Background

Based on worldwide GLOBOCAN estimates [1], there were an estimated 1.4 million diagnosed cases of colorectal cancer (CRC) and 693,900 CRC-attributable deaths in 2012. However, there are significant disparities in both incidence and mortality rates throughout the world. The increasing incidence in some Eastern European and Asian nations is believed to reflect changes in preventable CRC risk factors whereas decreasing mortality rates are generally occurring in high-resource nations which offer preventive screening and advanced treatment to their population. In anticipation of future global trends where colorectal cancer incidence is expected to rise coincidently with increasing prevalence of obesity and tobacco smoking, this manuscript will explore a microcosm of preventive screening resource distribution effects on geographic and population-based disparities set in the context of a relatively lower-resource state in a very high-resource nation. These insights may prove useful to the design and implementation of colorectal cancer screening programs [2].

CRC is the third-leading cause of cancer death in men and in women in the United States and the second-leading overall cause of cancer deaths in all Americans [3, 4]. In addition to its human toll, CRC imposes a tremendous economic burden; costs of CRC care are estimated at $14.14 billion for the year 2010, second only to breast cancer care [5]. As a disease affecting a significant proportion of the working population, the cumulative lost economic productivity associated with readily-avoidable CRC deaths will amount to as much as $33.9 billion by the year 2020 [6].

Colorectal cancer is highly preventable, as demonstrated by the landmark National Polyp Study which established that colonoscopic polypectomy reduces the incidence of colorectal cancer [7]. A follow-up study indicated that colonoscopy-guided removal of adenomatous polyps reduces death from colorectal cancer by 53 % [8]. Between 1990 and 2007, CRC mortality rates significantly decreased in all states except Mississippi, and the state-by-state decline in CRC mortality rates correlates with CRC screening compliance [9]. Based on data from the National Program of Cancer Registries (NPCR) for the year 2011, the US Centers for Disease Control and Prevention (CDC) listed Mississippi as the state with the highest CRC mortality rate for men and women of all races (http://apps.nccd.cdc.gov/uscs/cancersrankedbystate.aspx). The majority of gastrointestinal cancers have higher incidence rates in counties within the Mississippi Delta, one of the poorest and unhealthiest regions within the United States, than in non-Delta counties of Mississippi [10].

It has been projected that CRC mortality in the USA could be reduced by 50 % with currently available interventions [11], and deliberate public health action is being implemented in selected states to accomplish this goal [12, 13]. If the benefits of this national effort are to extend into Mississippi, one must first assess the CRC situation of this state. Approximately 37 % of Mississippians are of African ancestry, and regardless of the data source, numerous studies confirm worse CRC mortality rates prevail in African-Americans than in European-Americans [14]. Mississippi is a predominantly rural state with relatively low population densities, and studies in Utah, a state with even lower population densities, show that rural residents are less likely to be compliant with CRC screening than their urban counterparts [15]. However, the CDC lists Utah as the state with the lowest CRC incidence and mortality rates, so any obstacles imposed by a state’s rural nature are clearly surmountable. As of 2010, Mississippi has the highest percentage of adults with diagnosed diabetes (www.cdc.gov/diabetes/pubs/pdf/DiabetesReportCard.pdf), and a prior diagnosis of type 2 diabetes mellitus (TTDM) is a risk factor for CRC [16–18]. Mississippi has a very high prevalence of obesity [19], yet another risk factor for CRC [20, 21]. As of 2012, Mississippi has the highest poverty rate (24.2 %) in the United States [22], and increased CRC incidence and mortality rates are observed in counties and census tracts with high poverty rates [23–25]. Many low socioeconomic status (SES) attributes such as education [25] and insurance status [26] contribute to these disparities in CRC incidence and mortality. Yet even among insured residents, people living in lower SES neighborhoods are less likely to have had screening colonoscopies than those living in higher SES neighborhoods [27].

A geospatial approach to describing the landscape of colorectal cancer in Mississippi would be highly useful to better understand the factors which contribute to the overall poor CRC outcomes observed in the state. If, as is the case with breast cancer in Mississippi [28], geographic and population-based disparities exist not only with regards to disease outcomes but also with patterns of screening resource use, then such information would improve cancer control strategies and tactics. There are no extant public databases to describe the geographic distribution of CRC screening resources, such as licensed gastroenterologists or sites offering colonoscopies. The goals of this manuscript are to ascertain the geographic distribution of CRC screening resources, assess the extent of geographic and population-based disparities related to colorectal cancer, and determine whether CRC screening resource availability is a significant proximal factor in CRC outcomes in Mississippi.

## Methods

### Determination of colorectal cancer screening resources

Three telephone survey instruments were developed to verify the geographic location of colorectal cancer screening resources within Mississippi. Mississippi-licensed gastroenterologists were identified from the Mississippi State Board of Medical Licensure roster (July 25, 2013) of all state-licensed physicians. These gastroenterologists (or their staff) were contacted via telephone to (1) ensure the accuracy of their primary practice site address, and (2) verify they “currently practice gastroenterology within the state of Mississippi on a regular basis” as defined by practicing within Mississippi at least once each week, excluding vacation or holidays from consideration. Telephone surveys were conducted to contact the appropriate senior administrators of all licensed hospitals and ambulatory surgical facilities listed on the January 2013 Directory of Health Facilities published by the Bureau of Health Facilities Licensure and Certification of the Mississippi State Department of Health. These individuals were asked to (1) verify the accuracy of their facility address, and (2) verify whether the facility provided on-site colonoscopies. Telephone surveys were conducted over the period of July through August, 2013.

### Spatial data utilization and analysis

Spatial data were utilized in this study to: (a) identify the distribution of CRC healthcare resources in respect to the background data such as population, road network, county, etc. and (b) calculate areas within defined drive time distances from the CRC screening facilities. The verified street address of the healthcare resources were geocoded using ArcGIS 10.2.2 (Environmental Systems Research Institute, Inc.-ESRI, Redlands, California). Drive-time analysis was conducted to identify areas outside and inside of the 10-, 20- and 30-min drive-time distances to the CRC screening facilities; the irregular shape of these areas was a function of the existing road network and road characteristics. Drive-time distances for this study were calculated using quickest travel routes. Because 2013 CRC screening facility data were used, the estimated 2013 population and demographic data from ESRI Business Analyst were overlaid on the drive-time areas to identify the populations of interest and SES for inside and outside the drive-time distances.

### MCR colorectal cancer data

Colorectal cancer incidence and mortality data were obtained from the Mississippi Cancer Registry (http://mcr.umc.edu/) over the years 2006 through 2010 to provide a reliable “snapshot” of recent cancer statistics in a state dominated by low population density areas; 8392 diagnosed cases of CRC and 2993 CRC-attributed deaths were documented in Mississippi over this time period. Mississippi has a fairly stable population, as evidenced by U.S. Census data showing that the 0.3 % change in population in Mississippi from 2010 to 2011 was below the national average of 0.9 %, and population changes for individual Mississippi counties ranged from −3.0 to 3.2 %. Mississippi’s Behavioral Risk Factor Surveillance System (BRFSS) Public Health District survey report (http://msdh.ms.gov/brfss/index.htm) was the source of data on the use of colonoscopy and flexible sigmoidoscopy in Mississippians aged 50 and above were obtained from the District Reports for the years 2006, 2008 and 2010 (this question is not annually included in the BRFSS).

### Data analysis

Analysis was separately performed on three data sets: (1) the BRFSS Public Health District (PHD) level data set, (2) the Cancer registry county-level data set, and (3) the U.S. Census income data set. Histograms were constructed for all the variables in these three data sets to examine the normality of the distributions. Only the CRC screening variables of the county-level data exhibited skewed distributions. Correlation analysis was performed for both district-level and county-level data sets. The Spearman rank correlation coefficient was used in correlation analysis to accommodate the skewed distributions of the CRC screening variables of the county-level data. Though the normality was satisfied for the variables of the district-level data, the Spearman rank correlation coefficient was still used for consistency. The *P* values associated with correlation coefficients were reported against the null hypothesis of no association. Racial comparisons were also conducted for the district-level data. The paired T test was used to compare colorectal cancer outcomes and colonoscopy screening between Black and White Mississippi residents. The U.S. Census income data were characterized by clustering because the average incomes of the areas within and beyond 30-min drive to examination facility were obtained for one county. The U.S. Census income data were further complicated that some counties were entirely uncovered and the average incomes associated with beyond 30-min drive to examination facility for these counties were absent. The mixed model was used to analyze the clustered U.S. Census income data and address the aforementioned complexity. In the mixed models, the overall average incomes of counties were treated as random intercepts. The type of area, within or beyond 30-min drive to examination facility, was the fixed factor and the p values for comparing incomes between two areas were reported. All p values were two-sided and p values less than 0.05 were considered significant. Statistical analysis was performed using the software SAS (version 9.3, SAS Institute Inc).

No protected health information was collected for this study, which did not meet the definition of human subjects research.

## Results

### Results of district-level data

Based on the prior observation [28] of substantial population-based disparities in breast cancer mortality rates, mortality-to-incidence ratios, percentages of advanced-stage initial diagnoses of breast cancer, and the use of mammography, this study began by characterizing colorectal cancer attributes of Mississippi’s nine Public Health Districts (PHDs). Table 1 contains data derived from various sources which described colorectal cancer characteristics of the Public Health Districts. Mississippi’s Behavioral Risk Factor Surveillance System (BRFSS) data on colorectal cancer screening and Mississippi Cancer Registry colorectal cancer incidence, mortality and stage of disease at initial diagnosis are available as Black and White subsets; these subset data were compared via paired T test, and in most cases statistically-significant differences were noted between the White and Black subsets (Table 2). The four CRC characteristics significantly different between the Black and White subpopulations were: (1) age-adjusted colorectal cancer incidence rate (64.9 per 100,000 in Blacks vs. 50.5 per 100,000 in Whites; *P* < 0.0001); (2) age-adjusted colorectal cancer mortality rate (26.2 per 100,000 in Blacks vs. 17.5 per 100,000 in Whites; *P* = 0.0001); (3) colorectal mortality-to-incidence ratio (0.40 in Blacks vs. 0.35 in Whites; *P* = 0.0035); and (4) the percentage of Mississippians age 50 and older who reported they never had a sigmoidoscopy or colonoscopy (53.2 % in Blacks vs. 41.9 % in Whites; *P* < 0.0001). In all four of these characteristics, Blacks fared worse than Whites in Mississippi, confirming that population-based CRC disparities were pervasive throughout all nine PHDs in the state. Only one characteristic was not statistically different, and that was the percentage of advanced-stage colorectal cancers detected at initial diagnosis (52.9 % in Blacks vs. 52.3 % in Whites; *P* = 0.7926); it should be noted that a substantial percentage of CRCs are reported to the Registry with unknown staging, which partially masked the true values of this characteristic.

**Table 1:** Data characteristics of colorectal cancer and colorectal cancer screening resources in Mississippi’s Public Health Districts

Region	Practicing licensed GIs	Hospitals offering colonoscopy	Ambulatory surgical facilities offering colonoscopy	Age-adjusted CRC incidence rate (per 100,000; 2006-2010 MCR data)	Age-adjusted CRC mortality rate (per 100,000; 2006-2010 MCR data)	CRC mortality-to-incidence rate	% Advanced stage (regional + distant disease) at initial diagnosis	% (Age 50 +) reporting endoscopy	Population
Mississippi	104	61	26	54.49	19.79	0.3632	52.6	55.33	2,972,973
PHD 1	8	4	2	54.33	22.91	0.4217	53.5	54.87	319,959
PHD 2	16	5	5	53.36	20.33	0.3810	58.5	54.43	360,784
PHD 3	3	7	0	69.78	25.85	0.3704	47.4	45.80	216,708
PHD 4	6	4	1	54.88	18.72	0.3411	54.4	51.80	245,601
PHD 5	32	14	6	57.48	20.03	0.3485	51.2	60.60	640,418
PHD 6	6	7	2	53.86	18.69	0.3470	54.0	51.70	242,912
PHD 7	2	6	0	56.08	21.83	0.3893	53.4	54.53	174,798
PHD 8	15	6	4	50.90	16.73	0.3287	51.4	58.60	304,893
PHD 9	16	8	6	45.91	16.78	0.3655	51.0	59.17	466,900

The characteristics listed in this table include the number of practicing licensed gastroenterologists, the number of hospitals and ambulatory surgical facilities offering colonoscopy, the age-adjusted colorectal cancer incidence and mortality rates, the mortality-to-incidence ratios, the percentage of colorectal cancers initially diagnosed at advanced stage (regional + distant disease) and the number of residents according to 2010 US Census data. The percentage of residents (age 50 and older) reporting endoscopy was calculated as the average value from the 2006, 2008 and 2010 BRFSS of those who reported having received a colonoscopy or flexible sigmoidoscopy in each of Mississippi’s Public Health Districts (PHDs)

**Table 2:** Population-based disparities in colorectal cancer characteristics in Mississippi

Colorectal cancer characteristic	Black Mississippians	White Mississippians	Difference (Black–White)	*P* value
Statistically-significant health disparities				
% (Age 50+) reporting NEVER had a sigmoidoscopy or colonoscopy (average: 2006, 2008 and 2010 BRFSS)	53.2 %	41.9 %	11.3 %	<.0001
Age-adjusted colorectal cancer incidence rate (per 100,000; 2006–2010 MCR data)	64.9	50.5	14.4	<.0001
Age-adjusted colorectal cancer mortality rate (per 100,000; 2006–2010 MCR data)	26.2	17.5	8.8	0.0001
CRC mortality-to-incidence rate	0.40	0.35	0.05	0.0035
No discernible differences between groups				
% Advanced stage (regional + distant disease) at initial diagnosis	52.9 %	52.3 %	0.6 %	0.7926

Mississippi’s Behavioral Risk Factor Surveillance System (BRFSS) data on colorectal cancer screening and the Mississippi Cancer Registry colorectal cancer incidence, mortality and stage of disease at initial diagnosis were compared as Black and White subsets were compared via paired t test

**Fig. 1:**
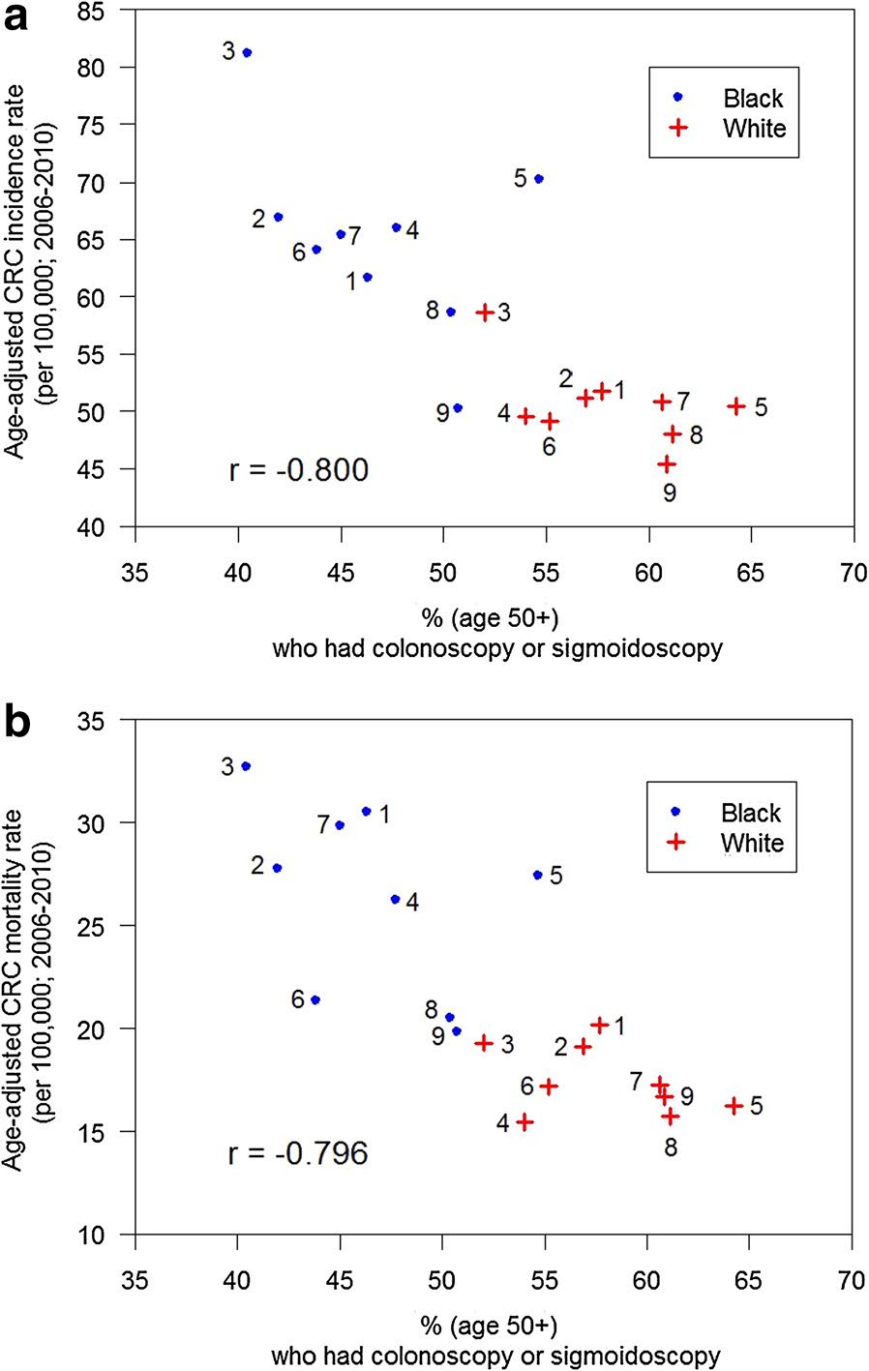
Public Health District patterns of colon endoscopy use are inversely correlated with adverse colorectal cancer outcomes. Mississippi BRFSS Public Health District survey report data (http://msdh.ms.gov/brfss/index.htm) from Black Mississippians (*blue dots*) and White Mississippians (*red crosses*) were obtained from the District Reports for the years 2006, 2008 and 2010, then averaged and plotted along the abscissa. *Panel* **a** The average age-adjusted CRC incidence rates from 2006 through 2010 for Black and White subsets were obtained from the Mississippi Cancer Registry for each of the nine Public Health Districts, then plotted on the ordinate. These* Black* and* White* subsets were subjected to a common Spearman rank correlation analysis to obtain a correlation coefficient of −0.800 (*P* < 0.0001). The *P* values associated with correlation coefficients were reported against the null hypothesis of no association. *Panel* **b** Average age-adjusted CRC mortality rates were obtained from the Mississippi Cancer Registry (http://www.cancer-rates.info/ms/index.php) for Black and White subsets from the years 2006 through 2010 for each of the nine Public Health Districts, then plotted along the ordinate. The Spearman rank correlation coefficient between these two variables was −0.796 (*P* < 0.0001)

Correlation analyses were then performed on Black and White subset data to determine whether the use of CRC screening via colonoscopy or sigmoidoscopy affected the primary CRC outcomes of incidence or mortality. A strong negative correlation (r = −0.800) was observed between the age-adjusted CRC incidence rate and percentage of individuals aged 50 years or older who reported having ever received a colonoscopy or flexible sigmoidoscopy (Fig. 1 panel a). Similarly, a strong negative correlation (r = −0.796) was also observed between age-adjusted CRC mortality rates and the percentage of screening-eligible individuals who reported having such endoscopic CRC screens (Fig. 1 panel b).

**Fig. 2:**
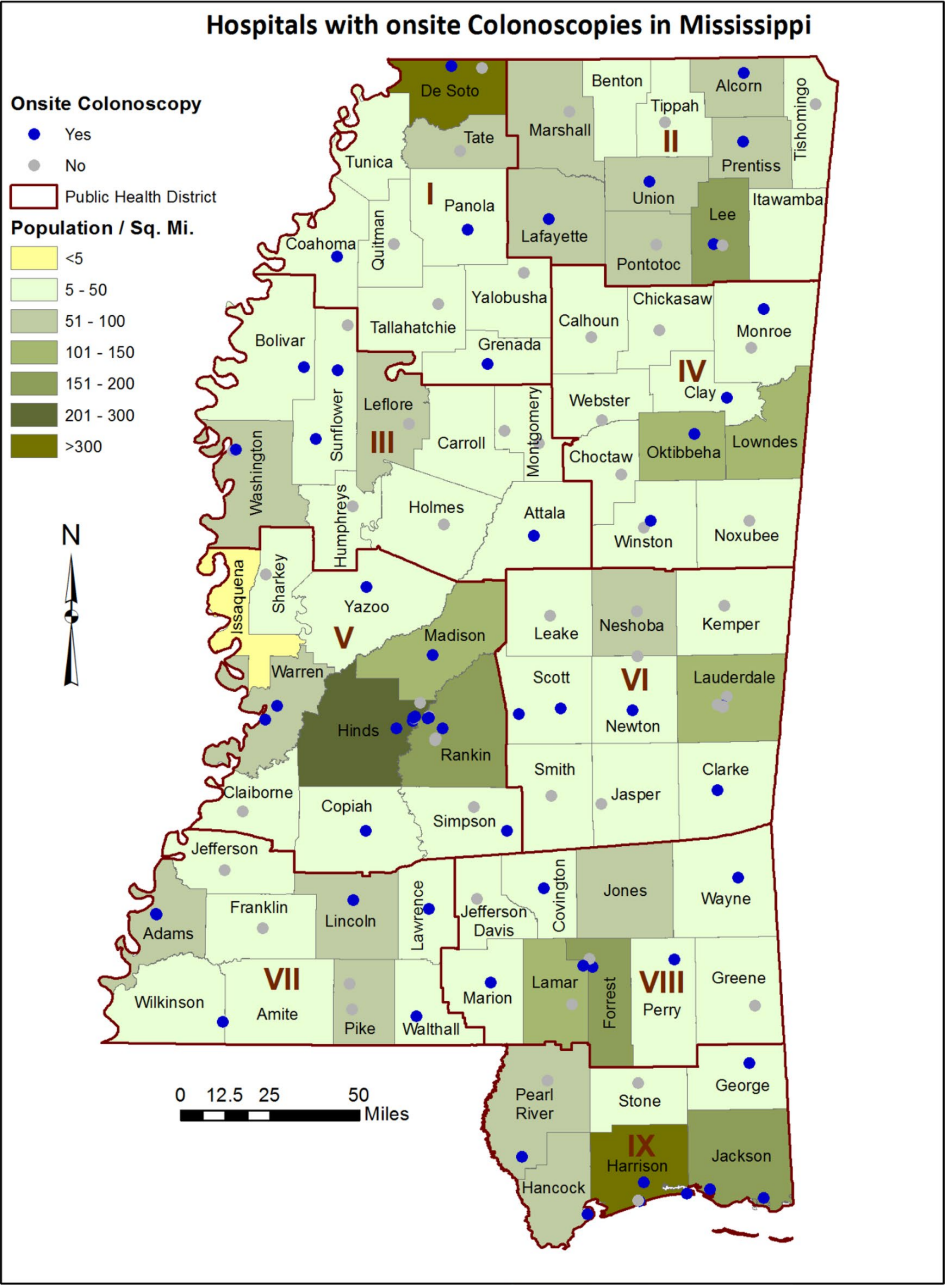
Map of colorectal cancer screening resources in Mississippi. As described in the “Methods”, telephone survey instruments were developed to identify the geographic address of the 61 hospitals (*blue dots*), as well as the 55 hospitals which do not offer on-site colonoscopies (*gray dots*). The hospitals and ambulatory surgical facilities are mapped by ZIP code. The population density (residents per square mile) is indicated for each county

**Fig. 3:**
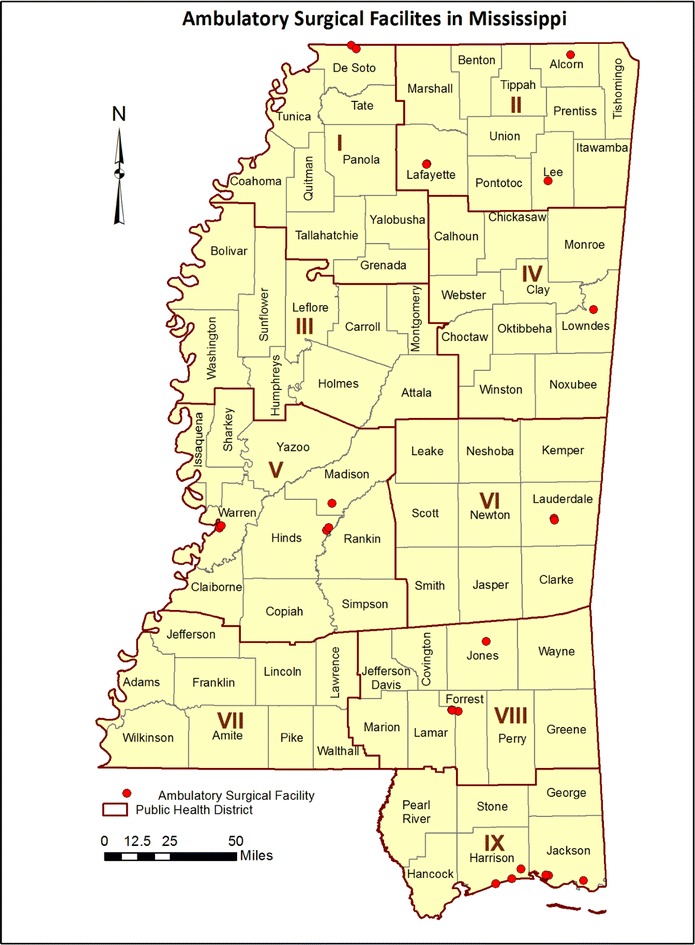
Map of colorectal cancer screening resources in Mississippi. As described in the “Methods ”, telephone survey instruments were developed to identify the 26 ambulatory surgical facilities (*red dots*) offering on-site colonoscopies in Mississippi. The hospitals and ambulatory surgical facilities are mapped by ZIP code

**Fig. 4:**
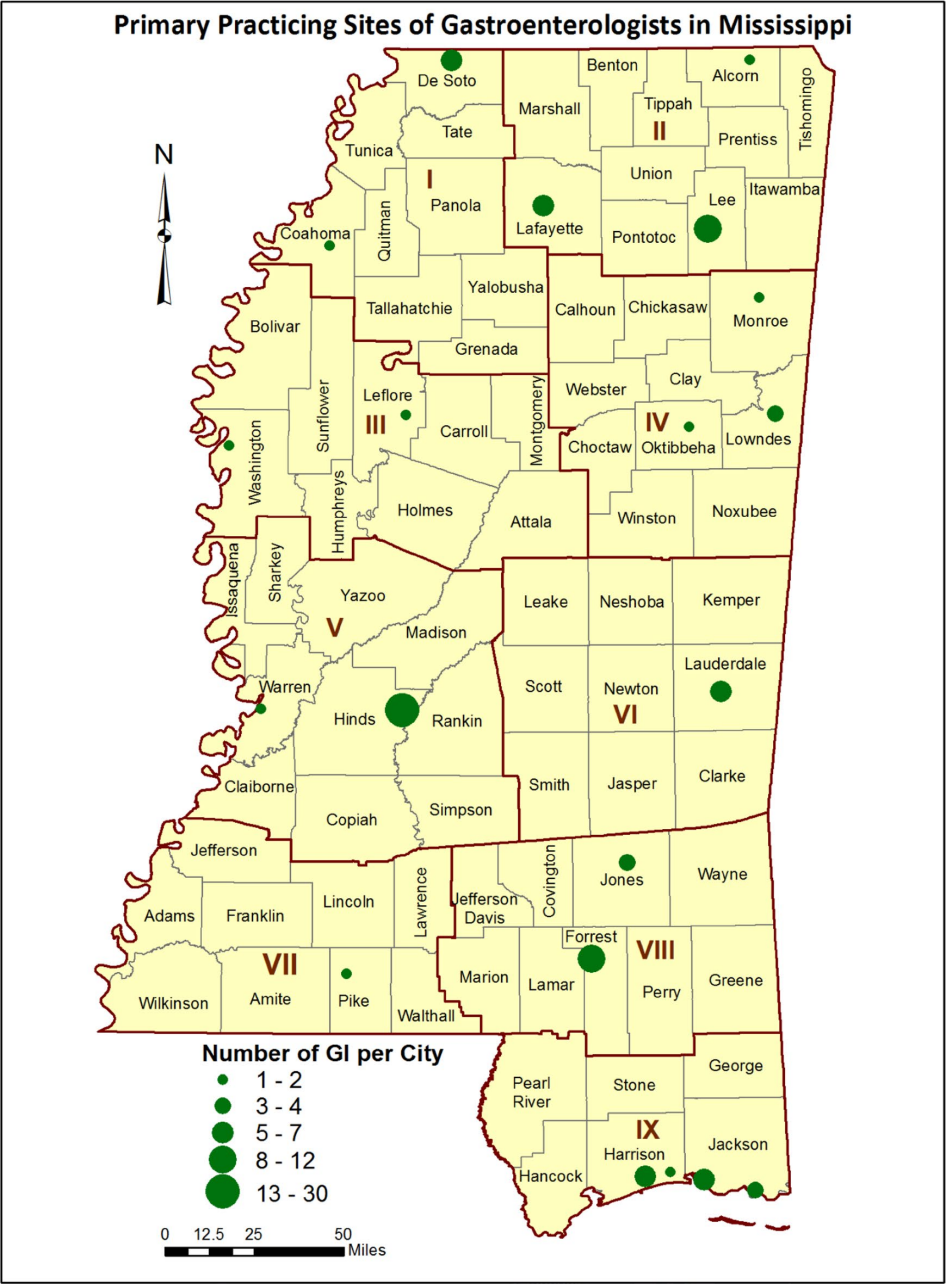
Map of colorectal cancer screening resources in Mississippi. The primary practice sites of 104 practicing licensed gastroenterologists are shown (*green dots*) according to the number of gastroenterologists per municipality. Five increasingly *larger green dots* are shown for municipalities which have 1–2, 3–4, 5–7, 8–12, and 13–30 gastroenterologists. Of the 21 Mississippi municipalities with gastroenterologists, 15 (71.4 %) have fewer than 5 gastroenterologists

An extremely broad range of endoscopic CRC screening rates were shown in Fig. 1, from a low of 40.4 % reported by Black Mississippians in Public Health District 3 to a high of 64.2 % reported by White Mississippians in Public Health District 5. The geographic distribution of endoscopic resources might contribute to such disparate results. While stool-based screens such as Fecal Immunohistochemical Tests (FIT) or high-sensitivity Fecal Occult Blood Tests (FOBT) are important early-detection screening methods, these resources are available through federally-qualified health centers, community health centers and primary care providers which are nearly ubiquitous throughout Mississippi, and are therefore unsuitable for geospatial analysis using the current study design. However, the availability of facilities to provide follow-up colonoscopy to confirm positive FOBT or FIT test results would be expected to affect the regional public health impact of these early-detection screens. Unlike mammography resources, which can be easily located via the US Food and Drug Administration’s Mammography Facility Database (http://www.accessdata.fda.gov/scripts/cdrh/cfdocs/cfMQSA/mqsa.cfm), no database currently provides a complete listing of sites which offer colonoscopies in Mississippi. The Bureau of Health Facilities Licensure and Certification of the Mississippi State Department of Health maintains a listing of all licensed health facilities in the state, and only two categories of health facilities (Hospitals and Ambulatory Surgical Facilities) are authorized to offer colonoscopies. Using the January 2013 Directory of Health Facilities as a primary data source, a telephone survey was conducted to determine which hospitals and ambulatory surgical facilities offer on-site colonoscopies and to verify the street addresses of these facilities. Figure 2 reveals the location of all hospitals which offer on-site colonoscopies (blue dots), as well as those hospitals which do not offer on-site colonoscopies (gray dots). Figure 3 shows the location of all ambulatory surgical facilities which offer on-site colonoscopies (red dots). Because it has been suggested that the accuracy and efficacy of colonoscopies performed by gastroenterologist endoscopists may be superior to those performed by non-gastroenterologist endoscopists [29, 30], the primary practice sites of gastroenterologists throughout Mississippi were also determined. Using a Roster of Licensed Physicians purchased from the Mississippi State Board of Medical Licensure, a telephone survey was conducted of all 178 physicians licensed in the specialty of gastroenterology as of July, 2013. Of these licensed gastroenterologists, 104 regularly practiced in Mississippi (defined as “practicing within Mississippi at least once each week, excluding vacation or holidays”), and the street addresses of their primary Mississippi practice sites were verified. These sites are shown (green dots) in Fig. 4; it should be noted that some gastroenterologists provide service at secondary practice sites, but secondary practice site data were not collected. At the time of the survey, only one Mississippi-licensed, out-of-state gastroenterologist regularly practiced in Mississippi; none of the other 66 Mississippi-licensed, out-of-state gastroenterologists did so. Four of the Mississippi-domiciled gastroenterologists were retired from practice; four others could not be contacted and their current telephone numbers could not be found, thus the gastroenterologist survey data were 97.7 % complete.

**Fig. 5:**
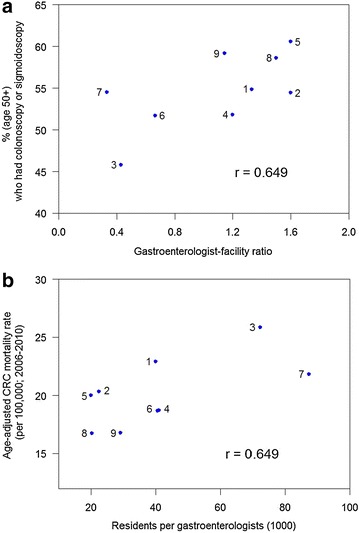
Relationships between colorectal cancer characteristics and CRC screening resources in Mississippi’s Public Health Districts. Data listed in Table 1 were subjected to a common Spearman rank correlation analysis. Two associative trends were observed. There is a positive association (R = 0.649, *P* = 0.058) between self-reported CRC screening rates and the ratio of gastroenterologists per facilities offering colonoscopies (*panel* **a**), and there is a comparable positive association (R = 0.649; *P* = 0.058) between colorectal cancer mortality rates and the number of residents per gastroenterologists (*panel* **b**)

Correlation analyses were then performed to ascertain whether any of these CRC screening resources affected CRC characteristics of Mississippi’s nine Public Health Districts. Although these were not as strong as the correlations presented in Fig. 1, and although they did not meet the threshold value for statistical significance (*P* ≤ 0.05), two interesting trends were noted. As shown in Fig. 5 (panel a), there was a positive associative trend (r = 0.649, *P* = 0.058) between self-reported CRC screening rates and the ratio of gastroenterologists per facilities offering colonoscopies. Thus, PHDs with fewer gastroenterologists than colonoscopy facilities (hospitals and ambulatory surgical facilities combined) tended to report lower rates of colonoscopy/flexible sigmoidoscopy usage. Conversely, PHDs with more gastroenterologists than colonoscopy facilities tended to report higher CRC screening rates. In addition, there was a positive associative trend (R = 0.649, *P* = 0.058) between colorectal cancer mortality rates and the number of residents per gastroenterologists (Fig. 5 panel b). Thus, PHDs with the lowest per-capita number of gastroenterologists tended to have the highest CRC mortality rates, and those with the highest per-capita number of gastroenterologists tended to have the lowest CRC mortality rates.

### Results of county-level data

**Fig. 6:**
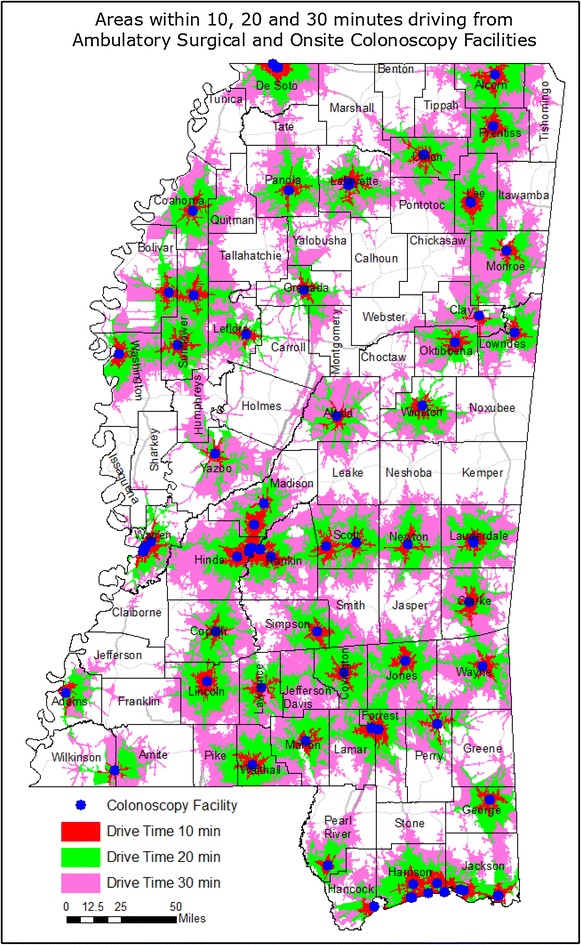
Geographic availability of colorectal cancer screening resources within defined driving times. This map displays the area that can be reached within a 10 min (*red*), 20 min (*green*) or 30 min (*purple*) automobile drive from each hospital or ambulatory surgical facility offering colonoscopies

**Fig. 7:**
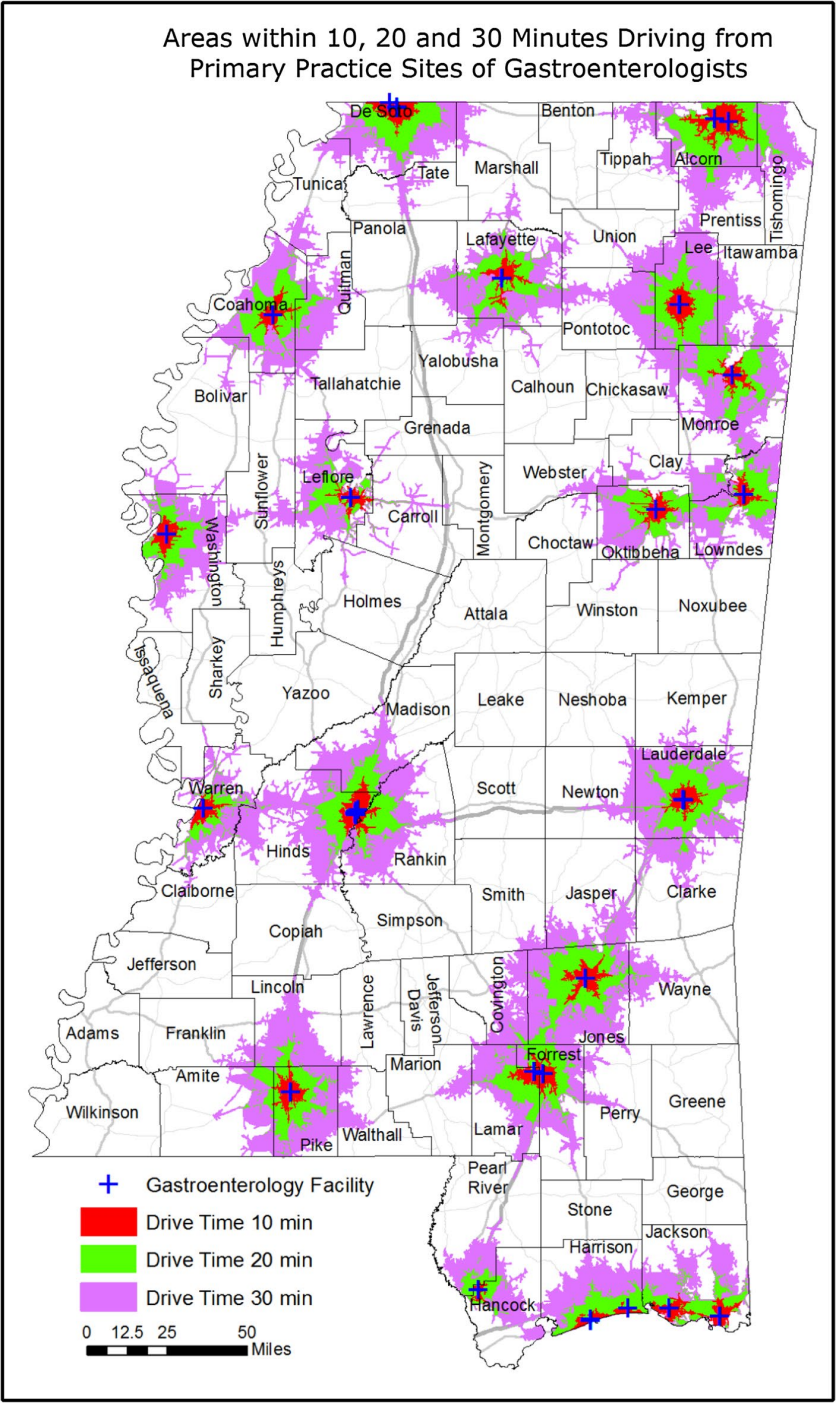
Geographic availability of colorectal cancer screening resources within defined driving times. This map displays the area that can be reached within a 10 min (*red*), 20 min (*green*) or 30 min (*purple*) automobile drive from the primary practice site of Mississippi-licensed gastroenterologists currently practicing within the state

With the exception of the BRFSS-derived CRC screening data which are only available at the Public Health District level, data granularity can be increased by evaluating CRC characteristics at the county level. Using the ArcGIS 10.1 software package (Environmental Systems Research Institute, Inc., Redlands, California), drive time areas were calculated in which residents could travel to a colonoscopy facility within 10, 20 or 30 min for each facility (Fig. 6). Drive time areas were also calculated for residents to travel to a gastroenterologist’s primary practice site within 10, 20 or 30 min for each practice site (Fig. 7). Geographically, 52 % of Mississippi is beyond a 30-min drive to a facility which offers colonoscopy, whereas 79 % of the state territory is beyond a 30-min drive to a gastroenterologist’s primary practice site. However, 83 % of the state’s population, with a mean per capita income of $20,680, live within a 30-min drive to a colonoscopy facility. In contrast, the 17 % of Mississippians who reside beyond this area have a mean per capita income of $16,894. Also, 62 % of the state’s population, with a mean per capita income of $21,680, live within a 30-min drive to a gastroenterologist’s primary practice site, whereas the remaining 38 % of Mississippians who live beyond this area have a mean per capita income of $17,307. These income values are collective state-wide population-based averages.

In correlation analysis, no county-level CRC health outcomes exhibited a dependency upon the length of time to drive to either a facility which offers colonoscopy or a gastroenterologist’s primary practice site. However, a strong county-level association existed between the incidence of colorectal cancer and the percentage of residents living below the poverty level (r = 0.461, *P* ≤ 0.000) and CRC incidence is negatively associated with median household income (r = −0.407, *P* < 0.001). At the county level, CRC incidence was positively associated with the prevalence of obesity (r = 0.388, *P* < 0.001) and the prevalence of type II diabetes mellitus (T2DM) (r = 0.304, *P* = 0.006), and negatively associated with the percentage of high school graduates (r = −0.384, *P* < 0.001). However, only an association between age-adjusted CRC mortality rates and T2DM prevalence remained significant at the county level (r = 0.376, *P* < 0.001).

Aside from direct CRC screening resources, the five county-level variables associated with colorectal cancer [prevalence of obesity, prevalence of T2DM, median household income, the percentage of adults (age 25 and older) who have graduated from high school, and the percentage of residents living below the poverty line] were strongly interrelated. The percentage of high school graduates was positively associated with median household income (r = 0.652, p < 0.001), and negatively associated with the percentage of residents living below poverty level (r = −0.565, *P* < 0.001), prevalence of obesity (r = −0.529, *P* < 0.001) and prevalence of T2DM (r = −0.453, *P* < 0.001). The percentage of residents living below poverty level was positively associated with the prevalence of obesity (r = 0.637, *P* < 0.001) and prevalence of T2DM (r = 0.466, *P* < 0.001), whereas the median household income was negatively associated with the prevalence of T2DM (r = −0.542, *P* < 0.001) and the prevalence of obesity (r = −0.519, *P* < 0.001). These results are summarized in Table 3.

**Table 3:** Spearman correlation coefficients for variables of county-level data (N = 82)

Variable 1	Variable 2	r	P
Age adjusted CRC incidence rate	% Below poverty level	0.461	<0.001
Age adjusted CRC incidence rate	Median household income	−0.407	<0.001
Age adjusted CRC incidence rate	Prevalence of obesity	0.388	<0.001
Age adjusted CRC incidence rate	Prevalence of type II diabetes mellitus	0.304	0.006
Age adjusted CRC incidence rate	% high school graduates	−0.384	<0.001
Age adjusted CRC mortality rate	Prevalence of type II diabetes mellitus	0.376	0.006
% High school graduates	Median household income	0.652	<0.001
% High school graduates	% below poverty level	−0.565	<0.001
% High school graduates	Prevalence of obesity	−0.529	<0.001
% High school graduates	Prevalence of type II diabetes mellitus	−0.453	<0.001
% Below poverty level	Prevalence of obesity	0.637	<0.001
% Below poverty level	Prevalence of type II diabetes mellitus	0.466	<0.001
Median household income	Prevalence of obesity	−0.519	<0.001
Median household income	Prevalence of type II diabetes mellitus	−0.542	<0.001

### Results of U.S. Census income data analysis 

The SES attributes including mean/median household income and per capital income were examined between within and beyond 30-min drives to colonoscopy facilities and to gastroenterologists’ primary practice sites at the county level (Tables 4, 5), and only the mean per capita income had a statistically-significant difference for areas defined for both examination facilities. The mean per capita income within 30-min drives to colonoscopy facilities was $17,797 (N = 82 counties) versus $17,141 (N = 82 counties) beyond 30-min (*P* = 0.049). The mean per capita income within 30-min drives to gastroenterologists’ primary practice sites was $18,334 (N = 68 counties) versus $17,294 (N = 82 counties) beyond 30-min (*P* = 0.016). Among the mixed models, the ratio of variance of random intercept to the residual variance ranged from 1.45 to 2.27. The magnitude of the variance of random intercept confirmed the necessity to include county effects as random intercepts.

**Table 4:** Incomes within and beyond 30-min drives to colonoscopy facilities

Variable	Within 30-min drives	Beyond 30-min drives	P value
Median household income	33,607	33,953	0.597
Mean household income	46,291	45,279	0.194
Per capital income	17,797	17,141	0.049

**Table 5:** Incomes within and beyond 30-min drives to gastroenterologists’ primary practice sites

Variable	Within 30-min drives	Beyond 30-min drives	P value
Median household income	35,058	33,889	0.279
Mean household income	47,370	45,572	0.083
Per capital income	18,334	17,294	0.016

## Discussion

We reported the univariate analysis results in this paper. Multivariate analysis of the county-level data was attempted. However, we failed to obtain any meaningful new results beyond what we have identified via univariate analysis. Several reasons explained the infeasibility of multivariate modeling. First, our study utilized existing databases in which only a limited number of factors were available. Second, the SES variables considered in the study are moderately correlated, reducing the stability of the potential multivariate models. Finally, Mississippi seems to be dominated by two distinct areas, the urban area with almost full CRC screening coverage and the rural area with little coverage. Lack of transitional areas makes it difficult to disentangle and quantify the separate effects of different factors. If our design and study can be replicated to a more diversified and larger geographic area, we would anticipate more profound effects as well as feasibility of multivariate modeling.

One cannot properly assess the burden of colorectal cancer in Mississippi without recognizing the problem of population-based disparities and the impact of community-level poverty. In all nine Public Health Districts, Black Mississippians have lower self-reported endoscopic CRC screening rates, higher CRC incidence rates and higher CRC mortality rates than White Mississippians. With a tangible commitment of strategic resources, Delaware has eliminated population-based disparities in CRC preventive screening and CRC incidence within a nine-year period [31], thus Mississippi’s problem is not intractable. However, additional geographic CRC disparities exist in Mississippi, in which Public Health District three fares worst in self-reported CRC screening rates, CRC incidence rates and CRC mortality rates. Notable geographic disparities also exist with respect to the availability of gastroenterologists, which ranges from a high of 20,013 residents per GI in PHD5 to a low of 87,399 residents per GI in PHD7, a 4.4-fold difference. Self-reported endoscopic screening rates are negatively correlated with CRC incidence and CRC mortality rates. When viewed from a regional perspective, there was a trend towards lower CRC mortality rates in Public Health Districts with higher gastroenterologists per capita, and there was a trend towards higher CRC screening rates in Public Health Districts with higher gastroenterologists per colonoscopy facility. These data are consistent with other published evidence that preventive CRC screening is the major proximal cause of lower CRC incidence and mortality in a public health setting. The effect of CRC screening resource availability was weaker at the county-level. No dependency on the length of drive time to either gastroenterologists or colonoscopy facilities could be detected over 10, 20 and 30 min intervals, which suggests that Mississippians with access to care are accustomed to long drive times to receive health services. At the county level, SES parameters and other preventable health conditions (T2DM and obesity) associated with population and geographic disparities had stronger associations with CRC outcomes than did the availability of CRC screening resources. Statistically-significant differences also exist in the per capita income of those who live closer to those resources than those who live farther away. Thus, endemic poverty is a major factor in the state’s CRC burden.

Mississippi’s rural nature complicates the statistical evaluation of existing problems in cancer control. To begin with, only 5 of Mississippi’s 82 counties have populations exceeding 100,000 residents, whereas 68 counties have populations less than 50,000 residents, a circumstance which intrinsically diminishes the statistical power of annual intra-state population studies. Thus, this study used CRC data from 2006 through 2010. Nearly half (36) of Mississippi counties lack hospices, which may skew the CRC mortality and mortality-to-incidence data if a significant proportion of residents moved from one county to another to receive end-of-life care. It is therefore reasonable that some of the correlations and associations observed at the Public Health District became less apparent at the county level due to regional relocation of patients between diagnosis and death. Further effort will be required to test this hypothesis and thereby improve rural public health data analysis for cancer control purposes.

This manuscript focused on the geographic distribution of CRC screening resources and their potential influence on CRC outcomes in Mississippi. Yet it was necessary to consider a few other factors associated with poorer CRC incidence and mortality rates, because piecemeal approaches to remedy individual factors have not succeeded in reducing geographic CRC disparities. CRC disparities continue to broaden as the CRC outcomes of high SES inhabitants of high SES communities improve while those of low SES inhabitants of low SES communities remain relatively static, a phenomenon which is common to many preventable diseases. A holistic model is needed to effectively reduce the disparate burden of preventable diseases, and Fundamental Causes Theory (FCT) provides one such model [32, 33]. How might FCT and informational diffusion rates affect CRC outcomes in Mississippi? Studies from various locations have indicated that the strongest predictor of whether an individual is compliant with CRC screening guidelines is whether a personal physician recommends a screening test to that person [34, 35]. Thus, the information concerning the importance of CRC screening must disseminate via personal physicians to the community before the benefits of increasing the regional availability of gastroenterologist-staffed colonoscopy facilities can have a definitive impact on CRC outcomes. Thus, the regional trends indicating an influence of gastroenterologists on screening behaviors may not merely reflect the specialized medical service they provide, they may also reflect the influence of regional gastroenterologists in disseminating CRC-related knowledge and awareness to surrounding primary care physicians, who then disseminate these benefits to the community.

The findings presented in this manuscript support the generation of a multi-step hypothesis to account for some of the geographic and population-based disparities in colorectal cancer observed in Mississippi. (1) Community-level wealth attracts gastroenterologists and ambulatory surgical facilities based on medical business principles. (2) Gastroenterology practice sites improve CRC screening awareness via medical marketing and improve awareness of CRC symptoms in the local medical community via dissemination of knowledge through peer-networks. (3) Improved CRC screening and CRC knowledge in the local medical community reduces CRC mortality. Further work and methodological improvements are required to rigorously test this hypothesis. If correct, the educational policy implications are subtle but profound, as it suggests that the health provider system in Mississippi must become more effective advocates of preventive CRC screening to their client communities, and that CRC prevention is not the sole responsibility of Mississippi’s health care consumers.

Academically, one can argue that additional data collection and analysis are required to test such hypotheses. Pragmatically, one must acknowledge that these observations are entirely consistent with the broad literature on colorectal cancer disparities. Data presented in Figs. 1, 2, 3 and 4 can be immediately applied to the development of regional interventions to improve CRC cancer prevention and control outcomes. If successful, such interventions may serve as models for developing nations where the availability of preventive screening resources have not yet kept pace with the rising incidence of colorectal cancer due to changes in lifestyle risk factors.

## Conclusions

Significant population-based and geographic disparities in CRC screening behaviors and CRC outcomes exist in Mississippi. The effects of CRC screening resources are related to CRC screening behaviors and outcomes at a regional level, whereas at the county level, socioeconomic factors are more strongly associated with CRC outcomes. Thus, effective control of CRC in rural states with high poverty levels requires both adequate preventive CRC screening capacity and a strategy to address fundamental causes of health care disparities.
